# Consistency of Integrated Management of Newborn and Childhood Illness (IMNCI) in Shire Governmental Health Institution in 2017

**DOI:** 10.1186/s13104-018-3588-y

**Published:** 2018-07-16

**Authors:** Hadgu Gerensea, Awoke Kebede, Zeray Baraki, Hagos Berihu, Teklay Zeru, Eskedar Birhane, Dawit G/her, Solomun Hintsa, Hailay Siyum, Gizenesh Kahsay, Gebreamlake Gidey, Girmay Teklay, Gebremeskel Mulatu

**Affiliations:** 1 School of Nursing, College of Health Science, Aksum University, Aksum, Ethiopia; 2 School of Public Health, College of Health Science, Aksum University, Aksum, Ethiopia; 3 Department of Midwifery, College of Health Science, Aksum University, Aksum, Ethiopia

**Keywords:** Consistency, Integrated Management of Newborn and Childhood Illness (IMNCI)

## Abstract

**Objective:**

In an effort to reduce infant mortality and morbidity, the World Health Organization and other technical partners developed the Integrated Management of Newborn and Childhood Illness (IMNCI). This study focuses on assessment of consistency and completeness of integrated management of neonatal and child hood illness in primary health care units.

**Results:**

A total of 384 cases were taken from 3562 cases both from young infant registration (under-2 month old) and child registration (2 months–5 year old). Out of 384 cases, 241 (62.8%) cases were correctly classified and 143 (37.2%) were incorrect classifications. Similarly 164 (42.7%) cases were treated correctly where as 220 (57.3%) treated incorrectly. Only 95 (24.7%) cases have given appropriate appointments where as 289 (75.3%) cases were appointed incorrectly. The overall consistency of IMNCI management is poor. Unless continuous follow up of and training was given, children are not treated as expected. More over using electronic method of IMNCI may alleviate the problem.

## Introduction

Integrated management of childhood illness (IMCI) is a globally proven, primarily community based strategy to improve child survival and is being implemented worldwide in countries with high burden of child mortality [1, 2].

In the past two decades childhood survival has shown significant improvement globally. This is due to the fact that many low and middle income countries have been implementing several strategies to improve child survival by targeting common causes of infant and child morbidity and mortality [3]. Despite these children fewer than five still die in large Numbers [4]. Improving quality of care in child health services will be essential for further substantial reductions in under-five child mortality [5].

IMNCI was pioneer into the health systems of over 100 countries that have high under five mortality with the strategy to change quality of child health care and decrease under-five mortality [6, 7].

Strict follow up to direction and algorithms is a complex activity. It is determined by contextual factors that may alter the health workers’ extrinsic and intrinsic capability and/or status to follow through these guidelines [8]. Up till now, researchers are not focusing about adherence to these approaches in under developed African countries, despite these countries being among the first that initiate IMCI into their general health plan of action [7].

Even though under five and neonatal mortality is decreased every year through different strategy primarily by IMNCI still know the reduction is not much as expected. This may be due to poor adherence and inconsistent use of IMNCI. So the present study was undertaken to evaluate the consistency of IMNCI on assessment, classification, treatment and follow up and this study will have base for the effective use of the guideline and shows missed diagnosis and not treated appropriately.

## Main text

### Study area

The study was conducted in Shire town which is located 1084 and 304 km far from the capital city of Ethiopia, Addis Ababa and the capital city of Tigray, Mekelle, respectively.

### Study period

The study was conducted from February to June in 2017 in shire Endasilasie town, Tigray regional state, north Ethiopia.

### Study design

Institution based cross sectional study design was used to address the objective from secondary data (IMNCI registration).

### Source population

All children under 5 years treated in public health institution using IMNCI guideline in the town.

### Study population

All sampled children who are under 5 years of age and treated in the health centers using IMNCI guide line.

### Inclusion criteria

Children under 5 years of age treated for neonatal and childhood illnesses only in the past 1 year will be included.

### Sample size determination

The sample size will be calculated using a single population proportion formula based on the following assumptions

$${\text{n}} = \left( {{\text{Z}}{\alpha /2}} \right)2{\text{p}}(1 - {\text{p}})({\text{d}})2$$

where n = minimum sample size required for the study, d = margin of error = 0.05, **Zɑ**/2 value of standard normal distribution (z = 1.96) with confidence interval of 95% and α is 0.05.P is taking by 50% or any prevalence of consistency and completeness of IMNCI study

$${\text{n}} = ({\text{z}}\upalpha/2)2{\text{p}}(1 - {\text{p}})({\text{d}})2 = (1.96)2 \cdot 0.5(1 - 0.5)(0.05)2 = 384$$

### Sampling procedures

To get the total sample we have used a systematic sampling technique from IMNCI registration every 10th interval was taken from the governmental health institution of shire both urban and rural. But the first sample was selected by simple random sampling (lottery method).

### Data collection procedures (instrument, personnel, data quality control)

Data were collected by reviewing registration using a format similar to IMNCI guide line which has five domains: assessing ill child, classifying for ill child, treating with medication, counseling care givers and need for referral.

### Data quality management

To assure high quality of the data, emphasis was given in designing data collection instrument and training on data collectors. Similarly the questioner format was pretested before the actual data collection period to modify the tool.

### Operational definitions

*Consistency of assessment with classification* how many cases are classified based on their assessment appropriately.

*Consistency of classification with treatment* how many cases are treated based on their classification appropriately.

*Correct classification and treatment* classification and treatments that fits based on IMNCI guideline regardless of heath professionals difference.

### Data analysis procedures

The collected data was checked for its completeness, consistency and accuracy before analysis. Data was analyzed and interpreted using SPSS version 21.

### Ethical considerations

Institution review board (IRB) of Aksum University, college of health science reviews the protocol to insure full protection of the rights study subjects. Following the approval by IRB, official letter of co-operation will write to respected study area. Data will treated confidentially and identify subject by number only.

## Result

### Disease profile of study participants

**Fig. 1:**
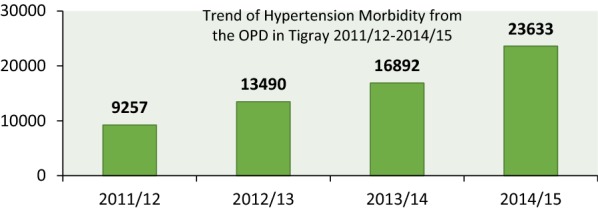
Disease profile of study participants

In all health institution nurses were primary used IMNCI guideline but in hospitals physicians were also involved. A total of 384 cases were taken from 3562 cases both from young infant registration (under-2 month old) and child registration (2 months–5 year old) 0.184 (47.9) cases were young infants where as 200 cases were children aged from 2 months up to 5 years. Of the total 196 (51.04%) were pneumonia cases and 105 cases were diarrhea. For further see Fig. 1.

### Consistency of IMNCI implementation

Pneumonia cases were 65.7% correctly clarified but 66.7% were incorrectly appointed or missed. From 40 cases of children who have fever only 10 (25%) and 7 (17.5%) children were classified and treated correctly.

Three cases (17.6%) were correctly managed for local bacterial infection, while 14 (82.7) were incorrectly classified, and managed for local bacterial infection. For further see Table 1.

**Table 1:** Consistency of assessment with classification, classification with treatment

S. no.	classification	Assessment with classification		Classification with treatment	
		Correct	Incorrect	Correct	Incorrect
1	Pneumonia	69 (65.7%)	36 (34.3%)	35 (33.3%)	70 (66.7%)
2	Diarrhea	81 (77.1%)	24 (22.9%)	64 (61%)	41 (39%)
3	Fever	10 (25%)	30 (75%)	7 (17.5%)	33 (82.5%)
4	LBW	14 (100%)	–	14 (100%)	–
5	LBI	3 (17.3%)	14 (82.7%)	3 (17.3%)	14 (82.7%)
6	Other	3 (100%)	–	3 (100%	–

*LBW* low birth weight, *LBI* local bacterial infection, *Other* ear infection, malnutrition

### Over all case management of IMNCI

**Fig. 2:**
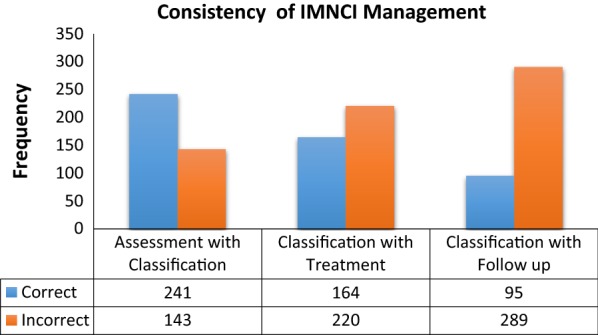
Consistency of IMNCI implementation

Out of 384 cases, 241 (62.8%) cases were correctly classified and 143 (37.2%) were incorrect classifications. Similarly 164 (42.7%) cases were treated correctly where as 220 (57.3%) treated incorrectly. Only 95 (24.7%) cases have given appropriate appointments where as 289 (75.3%) cases were appointed incorrectly. For further see Fig. 2.

## Discussion

Many scholars agreed that IMNCI guild line is the best tool for the accurate management of under 5 years children. Similarly it can avoid discrepancy among health institution and health professional but this research shows there is gap in the health professionals regarding the guide line or maybe there is negligence since many cases are classified and treated incorrect. But still different study shows IMNCI guide line has great effect on reduction of less than five mortality with the health professional inconsistency usage [9].

The study shows pneumonia is the leading cause of morbidity in under five children. This finding is similar with other study [10]. This may be due most children were classified by the IMCI algorithm based on the presence or absence of chest indrawing or fast breathing. One study shows 41% of the non physician health professionals have made misdiagnosis [11]. This may be the contributor for the magnitude of pneumonia.

But this finding was different with the study finding reviewed from 2010 to 2013 in Ethiopia in which diarrhea was the leading cause of morbidity [12]. this difference is may be related to the difference in qualification of health profession.

The study finding revealed that the proportion of IMNCI implementation was 54.2%. This is below standard level established (68%), by WHO and UNISEF [13]. This difference may be related with negligence of health professionals and may be also related to difference in qualification of health professionals.

Moreover, this study finding is quite higher than that of the study conducted in China [14]. The difference may be due to the policy difference in adherence to the guide line since WHO recommend using IMNCI for under developed countries like Ethiopia. Moreover, this study also shows higher implementation from the study conducted in Kenya which showed that, only 14% in the region [15]. This difference may be related to the study period gap and the study area difference in rural and urban as experienced health professionals are transferred in urban area and new health professional and untrained persons are recruited first in the rural area.

But this research finding is similar with the study conducted in five tertiary care hospitals of Karachi [16].

This study also contrary from the study conducted in Benin which showed performance of individual health workers varied greatly, from 15 to 88% of patients treated correctly, in accordance with the IMNCI guide lines [17]. This discrepancy is mostly due to the difference of individual on adhering of using IMNCI. Our study also shows that 62.8% consistency in assessing and classifying sick child which is better than that of study conducted in china which shows only 43.8% were correctly classified [14].

Treatment is about 42.7% consistent with classification, which still lower than other study finding [18] and much lower than study conducted in Benin which shows about 63.6% children treated according to IMNCI guideline [17]. This may be due lack of training, supportive supervision. Some authors noted a decline in performance and adherence rates depending on the time since the last IMCI (re-) training [19, 20], whereas others could not confirm these results [21, 22]. Similarly the updated guide line of 2016 is more different from the past in classification and some drugs are changed like cotrimoxazole to amoxicilline for treatment of pneumonia. But this finding is similar with study conducted in Pakistan which shows many children are treated incorrectly due to drug access problem and poor health professional’s knowledge on IMNCI [23].

But study conducted in Tanzania shows increase treatment use based on IMNCI. This difference could be due to the focus of the policy, intensive training and frequent supportive supervision [24]. More over this study finding is comparable with study conducted in India [18].

Regarding appointment we found only 24.7% consistency with its classification, which is low level, very low compared to WHOs 68% recommendation [13], and study conducted in Brazil (59%) [25]. But it is similar to study conducted in Bangladesh which says children fully assessed or correctly treated but there is problem in advising and follow up [26]. Moreover it is similar with study in Namibia, Kenya, Tanzania and Uganda which shows adherence rates to IMNCI is particularly low in non-hospital settings [27].

This study also found that, very low birth weight and neonatal jaundice, which are referral cases are highly consistent and complete 100% compared to other cases and much better than Bangladesh case which says none of the children classified for very low birth weight or prescribed correct medication at correct dose [26]. This may be related with few cases in our study.

## Conclusion

Despite the importance of IMNCI on reduction of under five mortality, the adherence rates for assessment and classification remained low. Most of the assessments are classified incorrectly. Similarly the correctly classified children are receiving inappropriate treatments.

In the light of these findings, special attention needs to be directed towards IMNCI training of all health staff, with particular emphasis on nurses since almost under-5 OPD are covered by nurses and should be consolidated with periodic re-training. The findings calls for continuing and increased efforts to improve the standard of child care within the framework of IMNCI. Moreover, using electronic method of IMNCI may alleviate the problem.

### Limitation

Since the study was conducted in specific area it should be repeated at national level to assess the outcome and long term impact of IMNCI and also to address the bottleneck problems.

## Abbreviations

IMNCI: Integrated Management of Newborn and Childhood Illness
WHO: World Health Organization
LBW: low birth weight
LBI: local bacterial infection
